# Long-Read epigenetic clocks identify improved brain aging predictions

**DOI:** 10.1101/2025.09.30.679553

**Published:** 2025-10-03

**Authors:** Spencer M Grant, Mary B Makarious, Melissa Meredith, Abraham Moller, Melissa Grant-Peters, Amy Hicks, Ajeet Mandal, Pavan Auluck, Hampton Leonard, Nicole Kuznetsov, Cory Weller, Xylena Reed, Miten Jain, Luigi Ferrucci, Mark R. Cookson, Mina Ryten, Mike A. Nalls, Kimberley J. Billingsley

**Affiliations:** 1. Center for Alzheimer’s and Related Dementias, National Institute on Aging and National Institute of Neurological Disorders and Stroke, National Institutes of Health, Bethesda, MD, USA; 2. Department of Clinical Neurosciences, School of Clinical Medicine, The University of Cambridge, Cambridge, UK; 3. DataTecnica LLC, Washington, DC, USA; 4. NIHR Great Ormond Street Hospital Biomedical Research Centre, University College London, London WC1N 1EH, UK; 5. Human Brain Collection Core, Division of Intramural Research, National Institute of Mental Health, NIH, Bethesda, MD, USA; 6. Department of Bioengineering, Northeastern University, Boston, MA, USA; 7. Longitudinal Studies Section, Translational Gerontology Branch, Intramural Research Program, National Institute; 8. Laboratory of Neurogenetics, National Institute on Aging, National Institutes of Health, Bethesda, MD, USA

## Abstract

Epigenetic clocks are widely used to estimate biological aging, yet most are built from array-based data from peripheral tissues of predominantly European-ancestry individuals, limiting generalizability. Here, we present aging clocks trained using GenoML, an automated machine learning platform for clinical and multi-omics data, on DNA methylation from Oxford Nanopore long-read sequencing. These models leverage over 28 million CpG sites across individuals of African and European ancestry. Our findings highlight the power of long-read methylation data for constructing accurate, ancestry-aware aging clocks and emphasize the importance of inclusive training datasets.

DNA methylation serves as a uniquely valuable modality for assessing biological aging due to its predictable age-associated patterns and its reflection of both genetic and environmental factors^1^. Consequently, epigenetic clocks, which are machine learning (ML) models that translate methylation data to determinations of biological age, have become increasingly relevant in tracking biological mechanisms associated with aging^1,2^. These clocks are often developed using variations of linear regression models, such as Elastic Net^2,3^, which aim to form an optimized linear combination of features to predict outcomes. These ML models are thus well-suited for epigenetic clocks due to their ability to combine DNA methylation reads across the genome, providing insights about an individual’s biological age based on molecular features.

Despite the promise they have shown, existing epigenetic clocks have several limitations. While existing models show strong performance in European cohorts, they fall short in their ability to generalize to non-European individuals^5^. Additionally, most clocks to date have been generated using array-based sequencing datasets, restricting the depth and quality of the epigenomic feature sets available to be used by ML models. Recent advances in long-read sequencing now enable high-resolution profiling of the methylome across the entire genome. Unlike array- or bisulfite-based methods, which capture <5% of CpG sites, Oxford Nanopore Technologies (ONT) long-read sequencing provides genome-wide methylation data at single-molecule resolution. For example, at the APOE locus, which has been established as prominent across neurodegenerative diseases^4,5^ with ancestry-specific genomic characteristics^6^, long-read data capture 33 times more CpGs than the 450K array, offering greater insight into densely methylated and functionally relevant regions^7^.

Here, we present epigenetic clocks that have been developed from long-read DNA methylation sequencing datasets including both European and African-ancestry individuals and using automated ML algorithm development. The data include both long-read methylation reads from prefrontal cortex tissue and clinical data, such as sex and postmortem interval (PMI), from the North American Brain Expression Consortium (NABEC) and the Human Brain Collection Core (HBCC). Given that age is the predominant risk factor for neurodegenerative disorders, brain samples are particularly valuable targets for epigenetic age modeling, reflecting methylation characteristics that are distinct from those observed in peripheral tissues^9^. The NABEC cohort includes harmonized methylation and clinical data from 187 individuals of European ancestry and the HBCC cohort includes harmonized data from 130 individuals of African ancestry. All participants have been determined to be neurologically healthy, making these cohorts suitable for generalizable, ancestry-specific epigenetic clocks. As shown in Supplementary Figure 1, the workflow includes long-read whole-genome sequencing, promoter-level DNA methylation aggregation, and predictive modeling using GenoML^10^, an open-source package for automated ML model development which competes numerous algorithms against each other and fine-tunes the best-performing algorithm to be then deployed on new data. GenoML’s competitive approach acknowledges that each dataset has unique structures and complexities, where a one-size-fits-all model would be inadequate. This ensures the final model is the best fit for the data at hand, both in performance and scalability, thus accommodating the complexity of long-read methylation data.

To establish baseline models, we developed epigenetic clocks that prioritize three sets of CpG sites (including 335, 816, and 760 CpGs, respectively) which were previously described in Lu *et al*. as being universally predictive of age across species and tissues^2^. We first generated six models, one model for each set of CpGs within each cohort. In line with prior studies, this yielded mixed results (0.442 ≤ R^2^ ≤ 0.881 on data withheld during model fitting), but with consistently more accurate predictions on participants from the NABEC cohort compared to HBCC (Figure 1). We then conducted a second benchmark analysis, similarly focused on the same loci, but using the aggregated methylation signal throughout the nearest promoter region to each locus in lieu of individual CpGs. This yielded 282, 608, and 590 distinct promoters, respectively, that passed quality control benchmarks for HBCC, and 283, 613, and 594 distinct promoters for NABEC. By accounting for signal from all CpG sites across each promoter, we aimed to reduce stochastic variability while preserving the functional significance of each of the previously-identified genomic regions. These promoter-based models performed substantially better than the CpG-based models (0.855 ≤ R^2^ ≤ 0.883 on withheld data), exhibiting improved accuracy for each set of CpGs in each cohort (Figure 2).

After establishing the predictive accuracy of long-read methylation data using known CpG sites, we sought to create epigenetic clocks based on unbiased identification of genomic regions within the NABEC and HBCC cohorts. Starting with all 29,598 promoters included in the Eukaryotic Promoter Database^11^, filtering steps were performed separately within each cohort to remove promoters that are highly correlated with each other and to keep only those which are significantly predictive of age. This yielded 3,260 and 5,996 promoters (of which 1,509 and 1,002 contributed to the performance of the final models) for the NABEC and HBCC cohorts, respectively, which were used as the final feature set for epigenetic clock development. These models predicted chronological age with high accuracy in withheld data from both the HBCC (R^2^ = 0.946) and NABEC (R^2^ = 0.901) cohorts, producing more accurate predictions than even the promoter-level models that depended on previously-reported loci (Figure 3). Intercepts and beta coefficients for each promoter from each of the two ancestry-informed models can be found in Supplementary Table 1. Feature importance plots using the Shapley Additive exPlanation (SHAP) package, which ranks promoters by their overall contributions to performance of the models, are shown in Supplementary Figure 2. Notably, and consistent with results reported elsewhere^12^, several promoters for genes across various protocadherin (PCDH) clusters were among the strongest predictors of age in NABEC, with a few also contributing to the final HBCC model. While the most important promoters are depicted, we note that the overall performance of the model is not strongly impacted by any small subset of promoters, but rather is democratically influenced by the contributions of many regions across the genome.

Altogether, the performance of each epigenetic clock workflow described here demonstrates several key considerations for designing accurate and equitable models. Aggregating methylation at the promoter level, rather than modeling individual CpGs, substantially improved prediction accuracy and cross-cohort generalizability. This can likely be attributed to the statistical noise from one CpG being smoothed out by neighboring signals that may have similar functional impacts. Moreover, the results were much more comparable between the NABEC and HBCC cohorts when using promoter-level data than CpG-level data, suggesting that this may have also helped mitigate technical and/or ancestry-specific differences in the methylation datasets. This method was achievable through the enhanced resolution of long-read sequencing data, highlighting its superiority in resolving epigenetic complexity. Finally, models that selected informative promoters through unbiased feature selection yielded the most accurate predictions, especially in HBCC, underscoring the need to account for population-specific epigenetic signatures in biological aging.

From the promoter-based models with unbiased feature selection, we identified genes that were both important for predicting age (|coefficient| > 0) and independently associated with age in each cohort (NABEC p < 7.7 × 10^−5^, HBCC p < 1.1 × 10^−4^). To avoid redundancy, genes associated with multiple significant promoters were included only once. Expression-weighted cell type enrichment (EWCE) analysis revealed that age-associated genes were enriched in mural and oligodendrocyte cell types across both cohorts, suggesting shared cellular signatures of aging in the brain tissues selected (Supplementary Figure 3). Additional enrichment was observed in neuronal populations, particularly in HBCC, and in astrocytes within NABEC. Gene ontology analysis indicated differential enrichment of genes involved in nervous system development, DNA binding, and transcriptional regulation pathways in HBCC compared to NABEC, and with many molecular function pathways significant across both cohorts (Supplementary Table 3, Supplementary Table 4, Supplementary Figure 4). These regions regulate developmental genes and maintain them in a poised state, particularly in progenitor and glial lineages. Taken together, these results suggest that age-predictive methylation signatures captured by long-read sequencing reflect both cell-type specific aging processes and epigenetic changes in polycomb-regulated, developmentally programmed regions of the brain.

Some limitations warrant consideration. The two cohorts were sequenced using different ONT chemistries (R9 for NABEC; R10 for HBCC), complicating direct comparisons since R10 more confidently calls methylation at the extrema (0% or 100% methylated) than R9^13^. Age distributions also differed: NABEC exhibited a bimodal distribution, while HBCC followed a more normal curve (Supplementary Figure 5). However, sensitivity analyses restricted to participants <80 years old yielded consistent results. In addition, the biorepositories used do not consistently capture real-world clinical information such as longitudinal or electronic medical record (EMR) data, and there is limited quantification of cause of death or comorbid illness burden, which may contribute to sample heterogeneity. As long-read methylation datasets grow, future clocks will benefit from larger sample sizes, harmonized sequencing protocols, and integration with comprehensive phenotypic data.

Together, these findings demonstrate that long-read methylation profiling enables accurate, ancestry-aware modeling of biological aging in the brain. Our results reinforce the need for inclusive, population-representative datasets and suggest novel cell type–specific pathways involved in aging. Finally, these workflows were developed in accordance with open science principles, with code publicly available at https://github.com/NIH-CARD/LongRead-Methylation-Clocks.

## Methods

### Long read DNA methylation data generation

We leveraged existing ONT WGS data generated from two postmortem brain cohorts: the North American Brain Expression Consortium (NABEC) and the Human Brain Collection Core (HBCC). Full details of the experimental protocol are available in^14^, but in brief, genomic DNA was extracted from prefrontal cortex tissue dissected from frozen brain samples for 130 neurologically normal individuals from the HBCC and 187 neurologically normal individuals from the NABEC^14^. ONT PromethION sequencing was performed on all samples using R9.4.1 flow cells basecalled with Guppy v6.12 for NABEC and R10.4.1 flow cells basecalled with Guppy v6.38 for HBCC; both cohorts included 5mC methylation in the basecalling command. Samples were sequenced until they reached at least 115GB of sequencing data. All samples were processed through the Nanopore Analysis Pipeline (Napu)^15^, wherein reads were aligned to GRCh38 with minimap2^16^ and methylation BED files were then produced using Modkit (https://github.com/nanoporetech/modkit) pileup to CpG motifs in the same reference. Methylation frequency at each CpG site was defined as the proportion of aligned reads carrying a methylated cytosine at that position.

### Methylation data processing and promoter-level aggregation

To evaluate the performance of existing epigenetic clocks using long-read methylation data, CpG sites corresponding to the Horvath Universal Mammalian Methylation clocks^2^ were identified. For any CpG sites from these clocks which were not captured in the datasets used here (of which there were 6, 16, and 19 for clocks 1, 2, and 3, respectively), the nearest CpG site was used in its place. Additionally, promoter annotations were obtained from the Eukaryotic Promoter Database^11^, where 60 basepair core promoter regions were expanded to 2 kb windows centered on transcription start sites to encompass a broader CpG context. CpG sites were filtered within each cohort to only include those with a coverage minimum of 5 reads for at least 95% of the participants in the respective cohort. Methylation was then averaged across all remaining CpG sites within promoter regions^17^ and limited to regions with a minimum of 10 CpG sites, yielding data for 28,592 promoters and 28,497 promoters in the NABEC and HBCC cohorts, respectively. Aggregating methylation frequencies across promoter regions reduces noise from individual CpG measurements, enhances statistical power, and enables interpretation of methylation at the level of regulatory elements.

Promoters were then filtered according to their correlation with other promoters and their independent associations with age. For each cohort, Pearson’s correlation was used to find correlation values for each pair of promoters, using batches of 1,000 consecutive promoters. Pairs with high correlation values (r > 0.9) were deemed to be redundant, and thus only the first promoter was kept for such pairs. Age was then regressed against all remaining promoters individually, adjusted by sex, PMI, and the first 20 methylation principal components, using ordinary least squares to determine which are most likely to be linked with aging. Only those with significant (p < 0.05) predictive power were kept.

These filtered and aggregated promoter-level methylation values were subsequently used as input features for machine learning model development.

### Comparisons with existing clocks

To compare with previously-reported methylation clocks, the CpG sites that were used to develop the three models described by Lu *et al*. were extracted from the long-read datasets used in this study. New models were developed according to the workflow described here using those individual CpG sites, as well as finding the nearest promoter to each of those loci and using those as inputs. As some pairs of CpG sites shared the same nearest promoter, each promoter was only included in the model once in the input dataset for each respective model.

### Statistical analysis and machine learning

For each clock model, the respective methylation data along with sex and PMI were used as inputs for machine learning model development using GenoML v1.5.4, using a 70/30 train/test split. Each model developed through GenoML included the munging, training, tuning, and testing steps in sequence using default parameters. All results shown represent fine-tuned model predictions on the withheld test data, with Elastic Net being selected by GenoML as the optimal algorithm for each of the clocks generated here.

### Gene set Enrichment Analysis

Genes of interest derived from both HBCC and NABEC datasets were functionally annotated with gene ontology (GO) terms using the gProfiler2 R package. Background gene lists consisted of genes included as inputs into models after filtering promoters for those associated with age or correlated with other promoters. Terms consisting of fewer than 10 or more than 2000 genes were removed, and p-values were corrected for multiple testing using the recommended g:SCS method. GO terms were reduced to parent terms using semantic similarity with a cut-off of 0.5, using the Rutils GitHub package (https://github.com/RHReynolds/rutils) to aid visualisation.

### Expression weighted cell type enrichment

We performed cell-type enrichment using Expression Weighted Cell Type Enrichment (EWCE, v. 1.4.0)^18^ to assess whether our genes of interest show significantly higher expression in specific cell types than expected by chance. This analysis was conducted on single-nucleus transcriptomes from 54,394 nuclei from the human dorsolateral prefrontal cortex (DLPFC) without neurological disorders. Preprocessing and annotation of this dataset is reported elsewhere^19^, and the function generate_celltype_data() from the R package EWCE was used to generate the celltype dataset.

Bootstrap gene lists controlled for transcript length and GC-content were generated with EWCE iteratively (n=10,000) using the “bootstrap_enrichment_test()” function. This function takes the inquiry gene list and a single cell type transcriptome data set and determines the probability of enrichment of this list in a given cell type when compared to the gene expression of bootstrapped gene lists, returning the probability of enrichment and fold-change of enrichment. P-values were corrected for multiple testing using the false discovery rate (fdr) method.

## Supplementary Material

Supplement 1

Supplement 2

Supplement 3

Supplement 4

Supplement 5

Supplement 6

Supplement 7

Supplement 8

Supplement 9

## Figures and Tables

**Figure 1.**
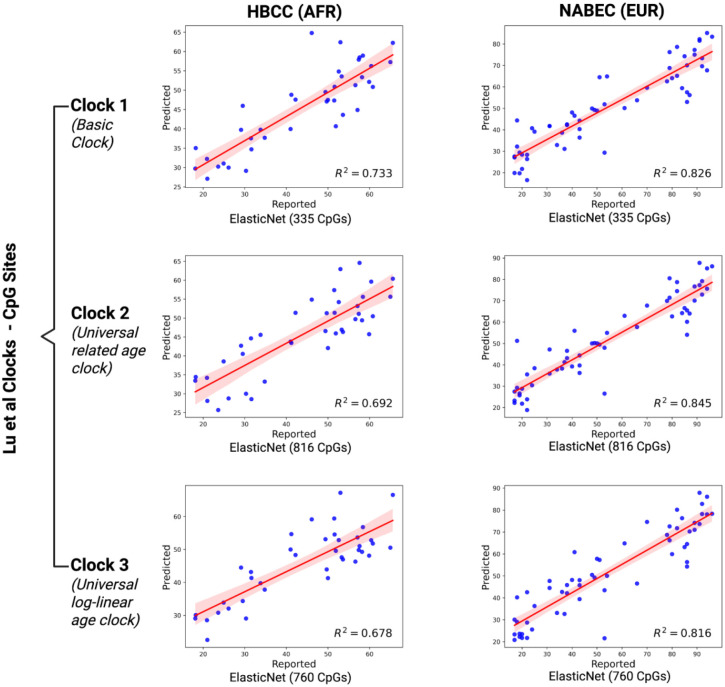
Ancestry-biased performance of existing epigenetic clocks in long-read brain methylation data. Age prediction models based on CpG sites from three established Horvath clocks were evaluated using long-read methylation data from postmortem brain samples. Models showed consistently higher accuracy in individuals of European ancestry (NABEC; right) compared to African and African admixed ancestry (HBCC; left), across all three clocks. Models were built using *GenoML* and evaluated across ancestrally diverse brain samples. Scatter plots show predicted versus reported age, regression fits, and R^2^ values for withheld data from each ancestry group and clock.

**Figure 2.**
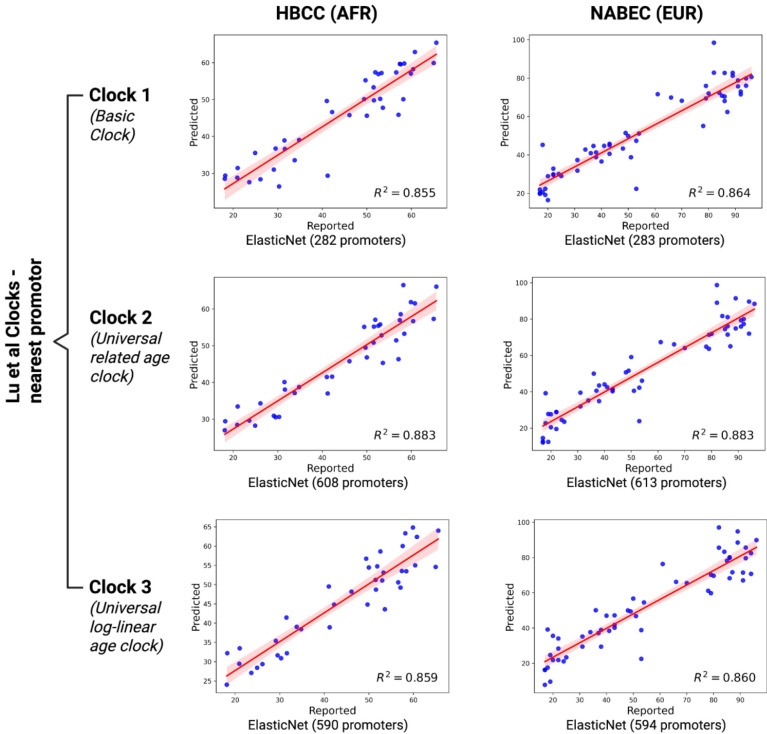
Improved age prediction using promoter-level methylation from long-read data. Age prediction models based on aggregated CpG methylation across promoters nearest to established clock CpG sites were evaluated using long-read methylation data from postmortem brain samples. Promoter-based clocks outperform models trained on individual CpG sites and demonstrate more cross-cohort generalizability. Models were built using *GenoML* and evaluated across ancestrally diverse brain samples. Scatter plots show predicted versus reported age, regression fits, and R^2^ values for withheld data from each ancestry group and clock.

**Figure 3:**
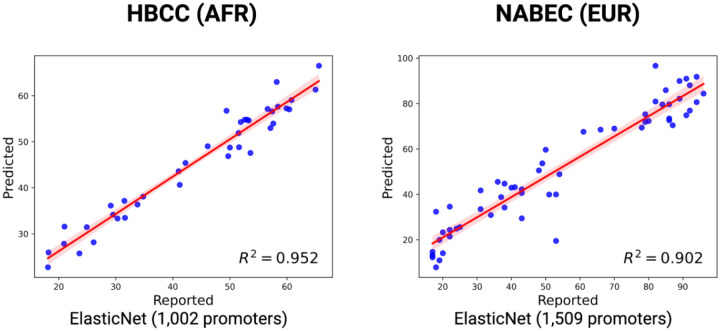
Unbiased, ancestry-informed age prediction using promoter-level methylation from long-read data. Age prediction models based on aggregated CpG methylation across 5,996 and 3,260 promoter regions for HBCC and NABEC brain samples, respectively, using unbiased selection criteria. Unbiased promoter selection demonstrated high accuracy in predicting age in both African and African admixed ancestry (HBCC) and European ancestry (NABEC) brain samples. Models were built using *GenoML* across ancestrally diverse brain samples. Scatter plots show predicted versus reported age, regression fits, and R^2^ for withheld data from each ancestry group.

## Data Availability

Human brain sequencing datasets are under controlled access and require a dbGap application (phs001300.v5) (phs000979.v4). Afterwards, the data will be available through the restricted AnVIL workspace.

## References

[1] Horvath S. DNA methylation age of human tissues and cell types. Genome Biol 14, R115 (2013). 24138928 10.1186/gb-2013-14-10-r115 PMC4015143

[2] Lu A. T. Universal DNA methylation age across mammalian tissues. Nat. Aging 3, 1144–1166 (2023). 37563227 10.1038/s43587-023-00462-6 PMC10501909

[3] Conole E. L. S., Robertson J. A., Smith H. M., Cox S. R. & Marioni R. E. Epigenetic clocks and DNA methylation biomarkers of brain health and disease. Nat Rev Neurol (2025) doi:10.1038/s41582-025-01105-7. 40533525

[4] Chen Z. Human-lineage-specific genomic elements are associated with neurodegenerative disease and APOE transcript usage. Nat Commun 12, 2076 (2021). 33824317 10.1038/s41467-021-22262-5 PMC8024253

[5] Belloy M. E., Napolioni V. & Greicius M. D. A Quarter Century of APOE and Alzheimer’s Disease: Progress to Date and the Path Forward. Neuron 101, 820–838 (2019). 30844401 10.1016/j.neuron.2019.01.056 PMC6407643

[6] Khani M. Biobank-scale genetic characterization of Alzheimer’s disease and related dementias across diverse ancestries. Nat Commun 16, 7554 (2025). 40813364 10.1038/s41467-025-62108-y PMC12354765

[7] Genner R. M. Haplotype-Resolved DNA Methylation at the Locus identifies Allele-Specific Epigenetic Signatures Relevant to Alzheimer’s Disease Risk. bioRxiv (2025) doi:10.1101/2025.07.01.662592. PMC13282175 42327426

[8] Cruz-González S. Methylation Clocks Do Not Predict Age or Alzheimer’s Disease Risk Across Genetically Admixed Individuals. bioRxiv (2024) doi:10.1101/2024.10.16.618588.

[9] Jacques M. DNA Methylation Ageing Atlas Across 17 Human Tissues. bioRxiv (2025) doi:10.1101/2025.07.21.665830.

[10] Makarious M. B. GenoML: Automated Machine Learning for Genomics. arXiv [cs.LG] (2021) doi:10.48550/ARXIV.2103.03221.

[11] Meylan P., Dreos R., Ambrosini G., Groux R. & Bucher P. EPD in 2020: enhanced data visualization and extension to ncRNA promoters. Nucleic Acids Res. 48, D65–D69 (2020). 31680159 10.1093/nar/gkz1014 PMC7145694

[12] Schmithorst V. Complex Regulation of Protocadherin Epigenetics on Aging-Related Brain Health. medRxiv (2024) doi:10.1101/2024.04.21.24306143.

[13] Genner R. Assessing DNA methylation detection for primary human tissue using Nanopore sequencing. Genome Res. (2025) doi:10.1101/gr.279159.124. PMC12047266 40054862

[14] Billingsley K. J. Long-read sequencing of hundreds of diverse brains provides insight into the impact of structural variation on gene expression and DNA methylation. bioRxivorg 2024.12.16.628723 (2024) doi:10.1101/2024.12.16.628723.

[15] Kolmogorov M. Scalable Nanopore sequencing of human genomes provides a comprehensive view of haplotype-resolved variation and methylation. Nat. Methods 20, 1483–1492 (2023). 37710018 10.1038/s41592-023-01993-x PMC11222905

[16] Li H. Minimap2: pairwise alignment for nucleotide sequences. Bioinformatics 34, 3094–3100 (2018). 29750242 10.1093/bioinformatics/bty191 PMC6137996

[17] Quinlan A. R. & Hall I. M. BEDTools: a flexible suite of utilities for comparing genomic features. Bioinformatics 26, 841–842 (2010). 20110278 10.1093/bioinformatics/btq033 PMC2832824

[18] Skene N. G. & Grant S. G. N. Identification of Vulnerable Cell Types in Major Brain Disorders Using Single Cell Transcriptomes and Expression Weighted Cell Type Enrichment. Front Neurosci 10, 16 (2016). 26858593 10.3389/fnins.2016.00016 PMC4730103

[19] Huuki-Myers L. Integrated single cell and unsupervised spatial transcriptomic analysis defines molecular anatomy of the human dorsolateral prefrontal cortex. bioRxiv (2023) doi:10.1101/2023.02.15.528722.

