# Supplementary material for: Long-Read epigenetic clocks identify improved brain aging predictions
Source: bioRxiv. 2025 Oct 3:2025.09.30.679553. Preprint. [Version 1] doi: 10.1101/2025.09.30.679553 (PMC12621889; doi:10.1101/2025.09.30.679553)
Supplement: Supplement 1 [file media-1.pptx]

## Slide 1
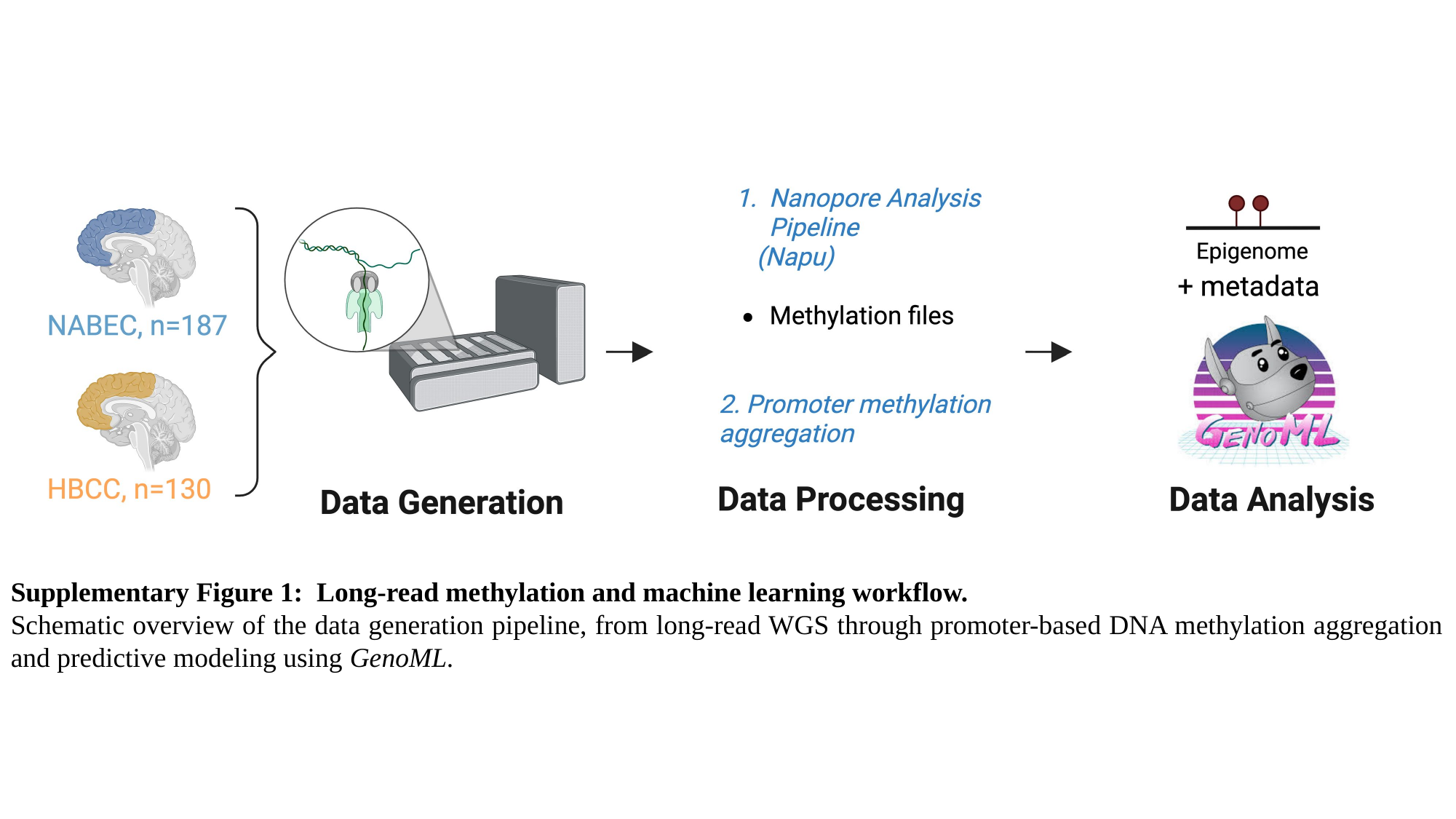

Supplementary Figure 1:  Long-read methylation and machine learning workflow.
Schematic overview of the data generation pipeline, from long-read WGS through promoter-based DNA methylation aggregation and predictive modeling using GenoML.
