# Supplementary material for: Long-Read epigenetic clocks identify improved brain aging predictions
Source: bioRxiv. 2025 Oct 3:2025.09.30.679553. Preprint. [Version 1] doi: 10.1101/2025.09.30.679553 (PMC12621889; doi:10.1101/2025.09.30.679553)
Supplement: Supplement 2 [file media-2.pptx]

## Slide 1
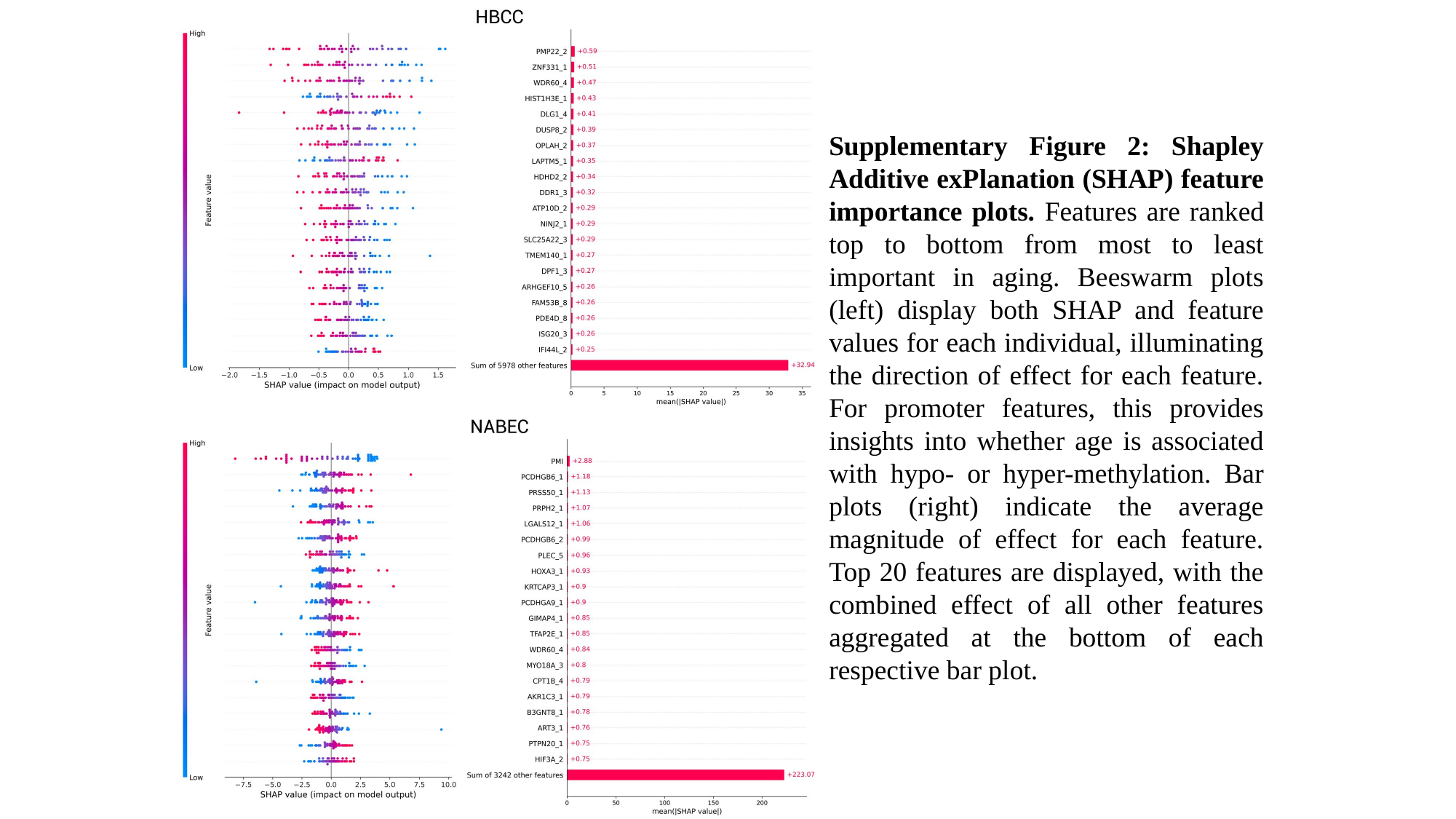

Supplementary Figure 2: Shapley Additive exPlanation (SHAP) feature importance plots. Features are ranked top to bottom from most to least important in aging. Beeswarm plots (left) display both SHAP and feature values for each individual, illuminating the direction of effect for each feature. For promoter features, this provides insights into whether age is associated with hypo- or hyper-methylation. Bar plots (right) indicate the average magnitude of effect for each feature. Top 20 features are displayed, with the combined effect of all other features aggregated at the bottom of each respective bar plot.
