# Supplementary material for: Long-Read epigenetic clocks identify improved brain aging predictions
Source: bioRxiv. 2025 Oct 3:2025.09.30.679553. Preprint. [Version 1] doi: 10.1101/2025.09.30.679553 (PMC12621889; doi:10.1101/2025.09.30.679553)
Supplement: Supplement 3 [file media-3.pptx]

## Slide 1
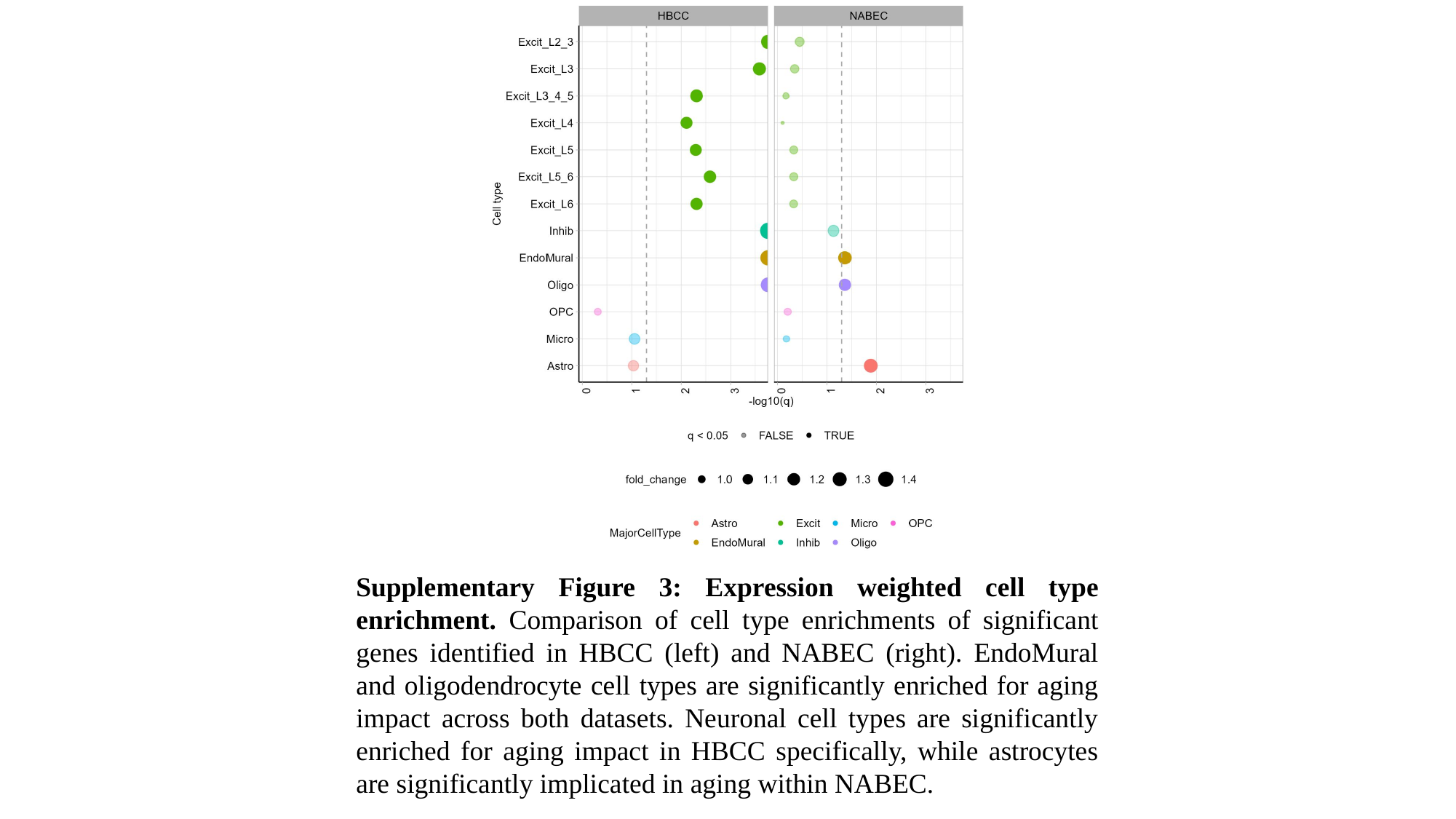

Supplementary Figure 3: Expression weighted cell type enrichment. Comparison of cell type enrichments of significant genes identified in HBCC (left) and NABEC (right). EndoMural and oligodendrocyte cell types are significantly enriched for aging impact across both datasets. Neuronal cell types are significantly enriched for aging impact in HBCC specifically, while astrocytes are significantly implicated in aging within NABEC.
