# Supplementary material for: Long-Read epigenetic clocks identify improved brain aging predictions
Source: bioRxiv. 2025 Oct 3:2025.09.30.679553. Preprint. [Version 1] doi: 10.1101/2025.09.30.679553 (PMC12621889; doi:10.1101/2025.09.30.679553)
Supplement: Supplement 4 [file media-4.pptx]

## Slide 1
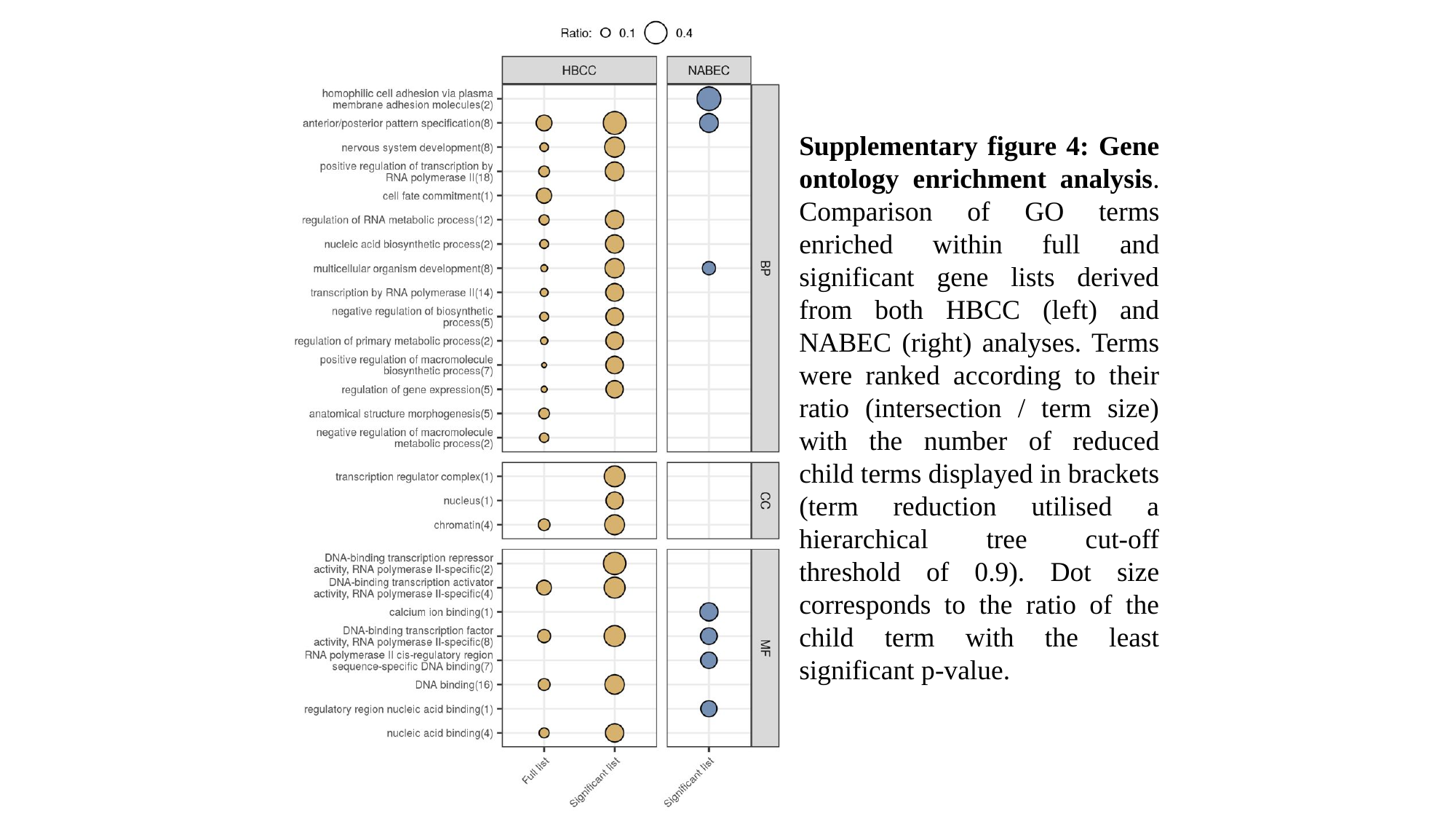

Supplementary figure 4: Gene ontology enrichment analysis. Comparison of GO terms enriched within full and significant gene lists derived from both HBCC (left) and NABEC (right) analyses. Terms were ranked according to their ratio (intersection / term size) with the number of reduced child terms displayed in brackets (term reduction utilised a hierarchical tree cut-off threshold of 0.9). Dot size corresponds to the ratio of the child term with the least significant p-value.
