# Supplementary figures and images for: Long-Read epigenetic clocks identify improved brain aging predictions
Source: bioRxiv. 2025 Oct 3:2025.09.30.679553. Preprint. [Version 1] doi: 10.1101/2025.09.30.679553 (PMC12621889; doi:10.1101/2025.09.30.679553)

## Slide 1
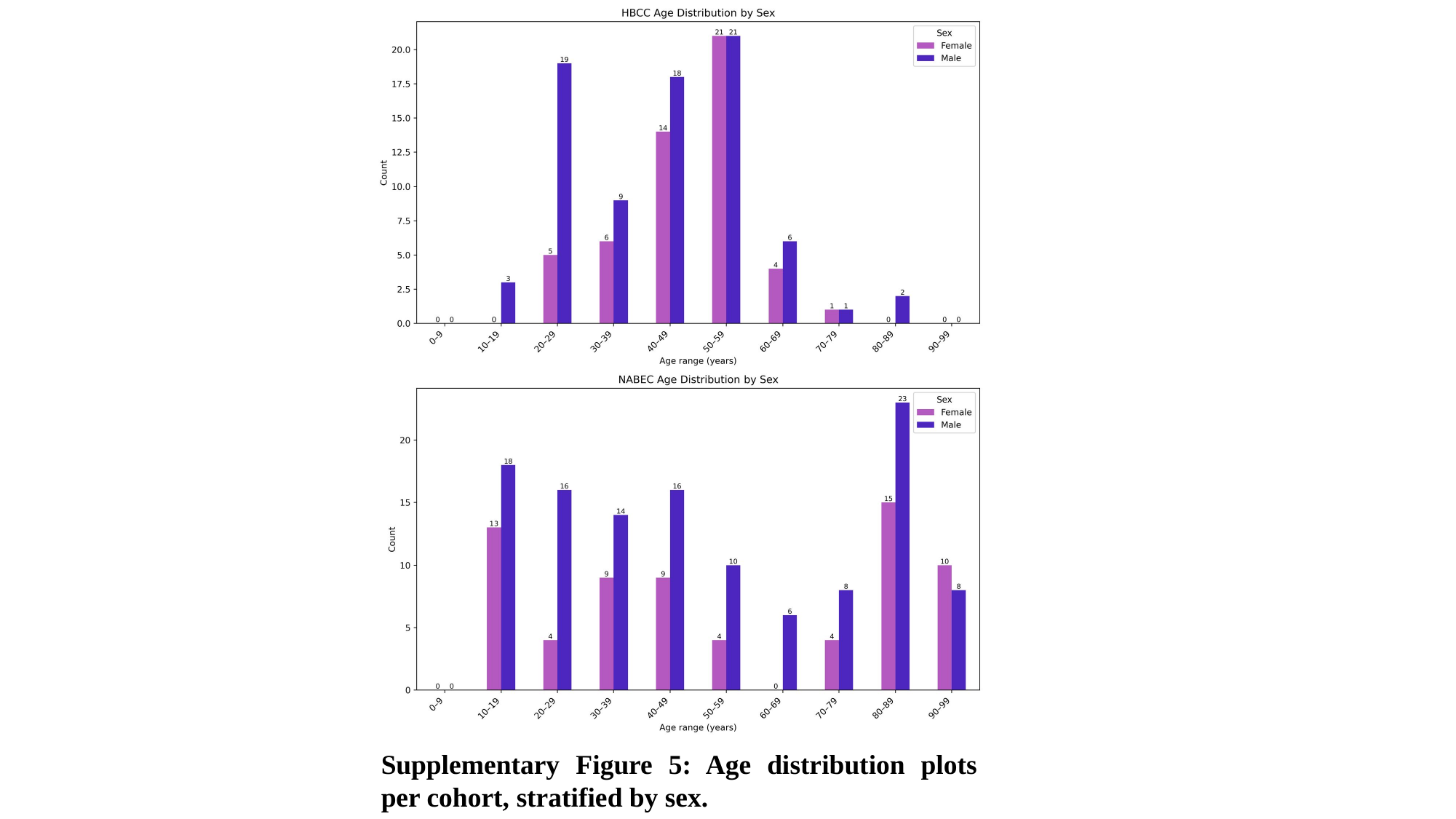

Supplementary Figure 5: Age distribution plots per cohort, stratified by sex.

Supplement: Supplement 5 [file media-5.pptx]
